# LC-HRMS/MS-based qualitative secondary metabolites profiling of *Leptadenia arborea* and evaluation of its antioxidant and hemostatic activities

**DOI:** 10.3389/fmolb.2026.1951196

**Published:** 2026-10-21

**Authors:** Beata Olas, Naglaa A. Rashidi, Antonietta Cerulli, Amal A. A. Mohamed, Bogdan Kontek, Natalia Sławińska, Karina Kalamat, Ibrahim B. Abdel-Farid, Sonia Piacente, Arafa I. Hamed

**Affiliations:** 1 Department of General Biochemistry, Faculty of Biology and Environmental Protection, University of Lodz, Łódź, Poland; 2 Phytochemistry Laboratory, Department of Botany, Faculty of Science, Aswan University, Aswan, Egypt; 3 Dipartimento di Farmacia, Università degli Studi di Salerno, Salerno, Italy

**Keywords:** blood platelets, flavonoid glycosides, *L. arborea*, LC-ESI/LTQOrbitrap/MS/MS, NMR spectroscopy, oxidative stress

## Abstract

**Introduction:**

*Leptadenia arborea* is an indigenous climbing plant whose phytochemical composition and biological properties remain poorly investigated. However, in regions of the Sahel and the Arabian Peninsula, *L. arborea* leaves, shoots, and young fruits are widely consumed as a highly nutritious green vegetable, spice, and soup ingredient. In addition, the leaves of *L. arborea* are highly valued for their dense nutritional content such as sugars, proteins, and amino acids, with remarkable levels of lysine, leucine, phenylalanine, and valine. For example, its leaves contain up to 18% protein by dry weight. They are also extremely rich in iron, calcium, potassium, and vitamin C.

**Methods:**

The present study employed a metabolomic approach based on LC-ESI/LTQOrbitrap-MS/MS to characterize the chemical composition of stalks and leaves extracts of *L. arborea*. The antioxidant activity, and antiplatelet effects of methanolic extracts from leaves (LaL) and stalks (LaS), together with three leaf-derived fractions [40% (LaLA), 60% (LaLB), and 80% (LaLC)] were also evaluated using various methods.

**Results:**

LC-ESI/LTQ-Orbitrap/MS/MS analysis enabled the tentative identification of fourteen metabolites, including flavonoids, a phenolic derivative, fatty acids, oxygenated fatty acids, and a diterpene glycoside. Five major flavonoids were isolated and unequivocally characterized by one- and two-dimensional NMR spectroscopy as apigenin-6,8-di-C-glucopyranoside (**1**), luteolin-7-*O*-β-glucopyranoside (**4**), apigenin-7-*O*-β-glucopyranoside (**6**), luteolin (**9**), and apigenin (**12**), confirming the LC-HRMS-based annotations. Antioxidant activity was assessed using an *in vitro* model of H_2_O_2_/Fe^2+^-induced oxidative stress in plasma and blood platelets. Among the tested samples, the 80% leaf fraction exhibited the strongest antioxidant activity, significantly reducing lipid peroxidation, protein carbonylation, and thiol oxidation in both plasma and blood platelets. Evaluation of thrombus formation using the Total Thrombus-formation Analysis System (T-TAS) showed that only the 40% fraction from the leaves at the two highest concentrations (5 and 50 μg/mL) significantly modulated blood platelet thrombus formation in human whole blood.

**Conclusion:**

These findings demonstrate that *L. arborea* is a rich source of bioactive flavonoids and other specialized metabolites with promising antioxidant properties, supporting its potential as a nutraceutical resource and providing a basis for future *in vivo* studies.

## Graphical Abstract

**Figure:**
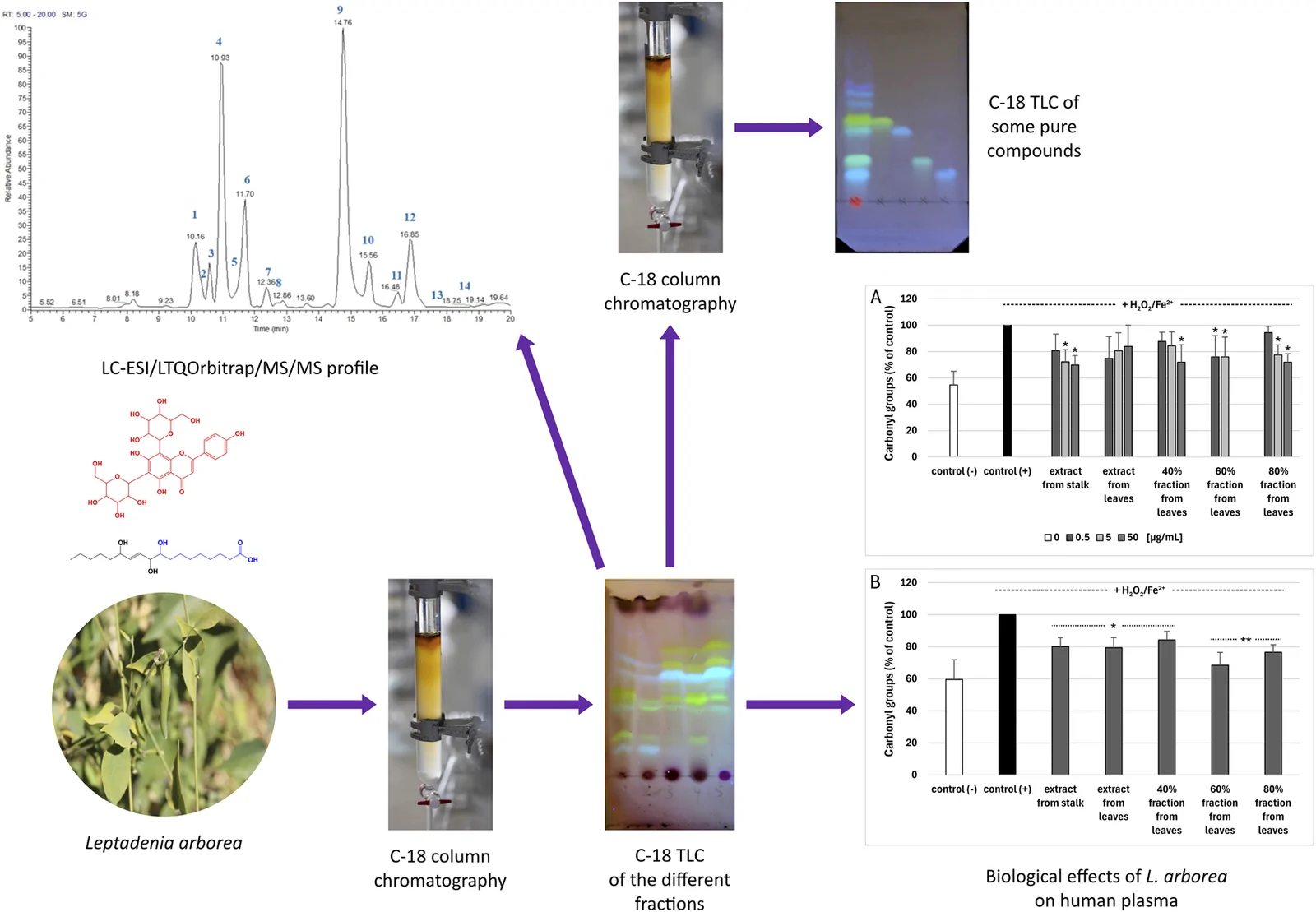

## 1 Introduction

The family Apocynaceae (formerly Asclepiadaceae) is a rich source of structurally diverse secondary metabolites, including flavonoid glycosides, cardiac glycosides, pregnane glycosides, alkaloids, terpenoids, steroids, and phenolic compounds, many of which exhibit remarkable pharmacological activities (Srivastav et al., 1994; Hamed et al., 2004; 2006; 2022; Perrone et al., 2008; Moustafa et al., 2009; Sonara and Saralaya, 2012; El-Fitiany and Khasawneh, 2023). Among the genera belonging to this family, *Leptadenia* comprises six species, namely *Leptadenia arborea* (Forssk.) Schweinf., *L. ephedriformis*, *L. hastata* (Pers.) Decne., *L. jazanica*, *L. pyrotechnica* (Forssk.), and *L. reticulata* (Retz.) Wight & Arn. (Deflers, 1896; Grubben, 2004; Schmelzer and Gurib-Fakim, 2008; Masrahi, 2015).

*Leptadenia arborea* (Forssk.) Schweinf (syn. *L. heterophylla*). is a medicinal plant shrub widely distributed throughout tropical Africa and the Arabian Peninsula (Bruyns et al., 2023). Known locally as “Maure”, it has long been used in traditional medicine for the treatment of snake bites, constipation, rheumatoid arthritis, gonorrhea, colic, fever, headaches, trypanosomiasis, syphilis, and neurological disorders, including migraine and mental illness (Doka and Yagi, 2009; Issa et al., 2018; Abdoon et al., 2019; Mohamed et al., 2020; Ngoupaye et al., 2020), antidepressant and anti-amnesic properties (Ngoupaye et al., 2020), as well as immunomodulatory (Abdoon et al., 2019) and anti-inflammatory activities (Osman et al., 2017). In addition, our previous studies revealed that *L. arborea* has sedative properties against migraine and decreases acetylcholinesterase activity (El-Hassan et al., 2003).

In regions of the Sahel and the Arabian Peninsula, *L. arborea* leaves, shoots, and young fruits are widely consumed as a highly nutritious green vegetable, spice, and soup ingredient. For example, its leaves and young shoots are boiled and chopped. Once cooked, they are often mixed with pulses like beans, or pigeon peas to create highly nutritious, protein-rich dishes. In addition, chopped leaves and flowers are added directly to traditional sauces and soups as a thickener and flavoring agent. Its young, slimy fruits are eaten as well. In Saudi Arabia, its branches and leaves are uniquely used in the cooking process of traditional dishes like Haneeth (slow-cooked goat meat) to add moisture and aromatic flavor to the dish (Ezike et al., 2016).

Notably, the leaves of *L. arborea* are highly valued for their dense nutritional content such as sugars, proteins, and amino acids, with remarkable levels of lysine, leucine, phenylalanine, and valine. For example, its leaves contain up to 18% protein by dry weight. In addition, they are also extremely rich in iron, calcium, potassium, and vitamin C (Souratié et al., 2025). However, extracts and other preparations from various parts of *L. arborea* are not approved by the U.S. Food and Drug Administration (FDA) for treating any medical condition (Souratié et al., 2025).

Despite the recognized phytochemical importance of the genus *Leptadenia*, the chemistry of *L. arborea* remains insufficiently explored. Previous studies reported only a limited number of constituents including α-amyrin, β-amyrin, stigmasterol, campesterol, some flavonoids such as apigenin, luteolin, and their derivatives, and a few phenolic compounds such as E-ferulaldehyde, leucanthemitol, pinoresinol, and syringaresinol, the latter displaying acetylcholinesterase inhibitory activity (El-Moghazy et al., 1981; El-Hassan et al., 2003; Elhameid et al., 2006; Elshikh et al., 2016). To address this gap, the present study employed a metabolomic approach based on LC-ESI/LTQOrbitrap-MS/MS to characterize the chemical composition of extracts from stalks and leaves of *L. arborea*. Comparative metabolomic analysis enabled the identification of 14 metabolites and revealed marked differences among the investigated samples. Guided by these results, major constituents were subsequently isolated and structurally characterized by NMR and MS.

Although the biological activity of various plants and their bioactive components, including phenolic compounds, has gained scientific attention for the last few decades (Aly et al., 2026; Botosoa and Shahidi, 2026; Demir et al., 2026; Lisjak et al., 2026), and it is well described, properties of *L. arborea* are definitely less recognized, especially in a context of oxidative stress and hemostasis, which are associated with different diseases (for example cardiovascular diseases (CVDs)). So far there is no data on the effect of *L. arborea* on these processes in human plasma and blood platelets. Because oxidative stress is an important factor in the modulation of hemostasis and in the progression of CVDs, for the first time, in this work, the biological potential of *L. arborea* was investigated through *in vitro* antioxidant and antiplatelet assays. The activities of the extracts, fractions, and the main flavonoids apigenin-6,8-di-C-glycoside (**1**), luteolin 7-*O*-β-glucopyranoside (**4**), apigenin 7-*O*-glucopyranoside (**6**), luteolin (**9**), and apigenin (**12**), were evaluated using biomarkers of oxidative stress in human plasma and blood platelets, as well as the Total Thrombus Formation Analysis System (T-TAS®). This integrated phytochemical and biological investigation provides the first metabolites characterization of *L. arborea* and expands current knowledge on its bioactive constituents and potential pharmacological applications.

## 2 Materials and methods

### 2.1 Chemicals and instruments

Methanol (isocratic grade), acetonitrile (LC-MS grade), formic acid (LC-MS grade), hexane, *n*-butanol, 5,5′-dithio-bis-(2-nitrobenzoic acid (DTNB), guanidine hydrochloride, hydrochloric acid (HCl), thiobarbituric acid (TBA), hydrogen peroxide, and sodium dodecyl sulfate (SDS) were from Merck (Darmstadt, Germany). Trichloroacetic acid (TCA), sodium chloride (NaCl), ethylenediaminetetraacetic acid (EDTA), ethanol, and ethyl acetate were purchased from POCH (Gliwice, Poland). All other reagents were purchased from commercial suppliers, including POCH (Gliwice, Poland), Merck (Darmstadt, Germany), and Chempur (Piekary Śląskie, Poland). The materials and reagents needed for the T-TAS measurements were from Bionicum Sp. Z o.o., (Warsaw, Poland). The other reagents were obtained from commercial distributors and were of the highest grade available.

The instruments used in this study included an UltiMate 3,000 ultra-high-performance liquid chromatography system (Dionex, Sunnyvale, CA, United States) interfaced with an electrospray ionization (ESI) source and a Q Exactive high-resolution mass spectrometer (Thermo Fisher Scientific, Waltham, MA, United States), Bruker Avance III 600 Ascend NMR spectrometer (Bruker, Berlin, Germany), UV-Visible Helios-α spectrophotometer (Spectrophotometer UV/Vis Helios alpha; Unicam, Cambridge, United Kingdom), SPECTROstar Nano Microplate Reader (BMG LABTECH, Ortenberg, Germany), and Total Thrombus-formation Analysis System (T-TAS) (Zacros, Tokyo, Japan).

### 2.2 Plant material

The aerial parts of *L. arborea* (Syn = *L. heterophylla*) were collected from Aswan Governorate, Egypt, in November 2023, during its flowering and fruiting stages. The plant was identified by Prof. Arafa I. Hamed according to Täckholm (1974), El-Hassan et al. (2003). Voucher specimens were deposited in the Herbarium of the Botany Department at the Faculty of Science, Aswan University, Egypt (No. 012020).

### 2.3 Extraction, fractionation, and preliminary phytochemical screening of *L. arborea*

The fresh plant materials were separated into two parts: leaves and stalks, and were air-dried at room temperature (36 ^°^C). The dried ground leaves (220 g) and stalks (125 g) were separately macerated with 80% MeOH for 48 h and extracted twice. The crude methanolic extracts were separately filtered and concentrated using a rotary evaporator at a temperature not higher than 60 °C, under reduced pressure, to obtain 25 g crude extract.

The 80% methanol–water (v/v) fraction obtained from the leaves of *L. arborea* (LaL, 12 g) was subjected to further fractionation. Briefly, LaL was dissolved in 10 mL of distilled water and loaded onto a preconditioned RP-18 column (6 × 10 cm, 60 μm C18, Baker). Elution was performed using a stepwise gradient of methanol in water, yielding five fractions: H_2_O (0% MeOH & 100% H_2_O, 1 g), 40% MeOH (LaLA, 3 g), 60% MeOH (LaLB, 3 g), 80% MeOH (LaLC, 2 g), and 100% MeOH (fatty fraction, 0.5 g). Each fraction was concentrated under reduced pressure, then lyophilized using freeze dryers (CHRIST-ALPHA 2–4 LD plus- Germany) and analyzed by reversed-phase thin-layer chromatography (RP-18 TLC; 20 × 20 cm, RP-18 F254s, Merck), using 40% acetonitrile as the mobile phase. Chromatograms were visualized by spraying with Liebermann reagent followed by exposure to ammonia vapor. Fractions LaLA, LaLB, and LaLC gave positive reactions with the detection reagents (aluminum chloride and ammonium hydroxyl vapor), indicating the presence of flavonoid derivatives and glycosylated compounds.

### 2.4 LC-ESI/HRMS/MS analysis

Qualitative metabolite profiling of extracts and the main fraction was performed by LC-ESI/LTQOrbitrap/MS/MS using an UltiMate 3,000 ultra-high-performance liquid chromatography system (Dionex, Sunnyvale, CA, United States) interfaced with an electrospray ionization (ESI) source and a Q Exactive high-resolution mass spectrometer (Thermo Fisher Scientific, Waltham, MA, United States). Prior to analysis, the extracts were dissolved in methanol–water (1:1, *v/v*) to obtain a final concentration of 1.0 mg/mL, and 5 μL of each solution was injected into the LC-HRMS system. Chromatographic separation was achieved on a Luna C18 column (150 × 2.1 mm, 5 μm; Phenomenex, Milan, Italy) using a flow rate of 0.2 mL/min and a mobile phase consisting of a combination of A (0.1% formic acid in water, *v/v*) and B (0.1% formic acid in acetonitrile, *v/v*). A linear gradient from 5% to 30% B in 2 min, from 30% to 95% B in 33 min, and held at 95% B for 5 min was used. The ESI source was operated in negative electrospray ionization mode with a capillary voltage of −48 V, a tube lens voltage of −176.47 V, and a source temperature of 280 °C. Nitrogen was employed as both sheath and auxiliary gas at flow rates of 15 and 5 arbitrary units, respectively. The mass range was from 120 to 1,600 m/z. Tandem mass spectrometric analyses were carried out using data-dependent acquisition (DDA), whereby the most abundant precursor ions detected in the full scan were automatically selected for fragmentation at a normalized collision energy of 30%. Instrument control and spectral acquisition were performed using Xcalibur software (Thermo Fisher Scientific). Data processing was carried out using Xcalibur Qual Browser. Feature detection and alignment were performed using a mass tolerance of <5 ppm, a signal-to-noise ratio >3, a minimum intensity of 1 × 10^4^, and a retention time window of ±0.2 min. Peak areas were normalized to the total ion current (TIC). Compound identification relied on accurate mass matching, isotopic pattern fit, and manual MS/MS spectral comparison with specialized literature on the Apocynaceae family.

### 2.5 Isolation and purification of compounds from *L. arborea* leaves fractions

The LaLA fraction (60% MeOH, 2.5 g) was subjected to chromatographic separation on a short RP-18 column (3 × 30 cm, LiChroprep® RP-18, 25–40 μm, Merck), previously conditioned with methanol and equilibrated with water. Elution was carried out using acetonitrile/water (20:80, v/v) as the mobile phase, and 5 mL fractions were collected. All subfractions were monitored by RP-18 thin-layer chromatography (C18-TLC) using 40% acetonitrile as the developing solvent. Based on TLC profiles, fraction LaLA-1 was selected for further purification. This fraction was concentrated under reduced pressure and re-chromatographed on a second RP-18 column (2 × 30 cm, LiChroprep® RP-18, 25–40 μm, Merck) under the same chromatographic conditions. Elution with 20% acetonitrile afforded compound **1** (20 mg) and compound **4** (35 mg) in pure form.

The LaLB fraction (40% MeOH, 2.5 g) was chromatographed on a short RP-18 column (3 × 30 cm, LiChroprep® RP-18, 25–40 μm, Merck), previously conditioned with methanol and equilibrated with water. Elution was performed with 20% acetonitrile, and the resulting fractions were analyzed by C18-TLC developed with 40% acetonitrile. Fractions LaLB-4, LaLB-5, and LaLB-7 were obtained, together with compound **6** (85 mg) in pure form.

Fraction LaLB-4 was concentrated and further purified on a short RP-18 column (2 × 30 cm, LiChroprep® RP-18, 25–40 μm, Merck). Following equilibration with water after methanol conditioning, elution with 20% acetonitrile yielded compound **9** (18 mg). Fraction LaLB-5 was similarly concentrated and re-chromatographed on an RP-18 column under identical conditions, affording compound **12** (57 mg) in pure form.

### 2.6 NMR analysis

Structural characterization of the isolated compounds was performed by NMR spectroscopy. NMR spectra were recorded in methanol-*d*_4_. All measurements were performed on a Bruker Avance III 600 Ascend NMR spectrometer (Bruker, Germany) operating at 600 MHz equipped with a 5-mm TCI CryoProbe at 300 K. Spectral processing and analysis were carried out using TopSpin 3.2 software.

### 2.7 Preparation of stock solutions (extracts and fractions) and pure compounds for bioassays

The extracts, fractions, and chemical compounds isolated from extracts/fractions were dissolved in 50% DMSO (dimethyl sulfoxide, a universal solvent for many different plant substances) and stored at −20 °C. The pure compounds were also dissolved in 50% DMSO. The final concentration of DMSO in the tested human whole blood, plasma and blood platelets was kept below 0.5% (*v*/*v*) to eliminate the effect of the solvent on the experiments (Jantan et al., 2012; Jayakumar et al., 2017). The final concentrations of the extracts/fractions in the samples ranged from 0.5 to 50 μg/mL. The final concentration of pure chemical compounds (isolated from extracts/fractions) in the samples was 5 μg/mL.

### 2.8 Blood, plasma, and blood platelet samples

Human whole blood was collected at “Diagnostyka” blood collection center (Brzechwy 7A, Lodz, Poland). All donors (aged 19–24) were non-smoking, healthy volunteers who did not drink alcohol or take medication, including anti-platelet and antioxidant supplementation, for 2 weeks before blood collection.

Peripheral blood was drawn into tubes with benzylsulfonyl-D-Arg-Pro-4-amidinobenzylamide (BAPA) (for T-TAS) or with citrate/phosphate/dextrose/adenine (CPDA) anticoagulant (for the remaining assays). The analysis of blood samples was performed according to the guidelines of the Helsinki Declaration for Human Research. An informed consent form was signed by each participant 1 day prior to blood collection. Procedures were conducted with the consent of Bioethics Committee at the University of Łódź (2/KBBN-UŁ/III/2014). Blood for the T-TAS assay was used within 2 h after collection.

Blood platelets were isolated from fresh whole blood by differential centrifugation. The procedure was described in earlier papers (Jędrejek et al., 2017; Sławińska et al., 2023; 2024; 2025). In every experiment, blood, plasma, or blood platelets were incubated for 30 min at 37 °C with *L. arborea* extract or the tested fractions (40%, 60% and 80%) at final concentrations of 0.5–50 μg/mL. The final concentration of pure chemical compounds (isolated from extracts/fractions) in plasma and blood platelets was 5 μg/mL. Protein concentration in samples was measured by bicinchoninic acid (BCA) method (Smith et al., 1985).

### 2.9 Lipid peroxidation measurement (in blood platelets and plasma)

Lipid peroxidation was measured based on the concentration of thiobarbituric acid-reactive substances (TBARS) as described earlier (Sławińska et al., 2023; 2024). Plasma or blood platelets were incubated with extracts/fractions or pure compounds and an oxidative stress inducer (4.7 mM H_2_O_2_/3.8 mM Fe^2+^/2.5 mM EDTA) for 30 min at 37 °C. A negative control (without H_2_O_2_/Fe^2+^ or extracts/fractions/pure compounds) with 0.9% NaCl was also set up. After the incubation, equal amounts of 15% TCA in 0.25 M HCl and 0.37% TBA in 0.25 M HCl were added to the samples; the samples were mixed and placed in a 100 °C thermoblock for 15 min. Next, the samples were cooled and centrifuged (10,000 × g, 15 min, 18 °C). The absorbance of the supernatant was measured using a SPECTROstar Nano Microplate Reader (BMG LABTECH, Germany) at 535 nm. TBARS concentration was calculated with a molar extinction coefficient (ε = 156,000 M^−1^ cm^−1^). The results were expressed as nmol/mL plasma or nmol/10^8^ blood platelets.

### 2.10 Protein carbonylation measurement (in blood platelets and plasma)

Protein carbonylation was measured with a method employing 2,4-dinitrophenylhydrazine (DNPH) as described earlier (Sławińska et al., 2023; 2024). Plasma or blood platelets were incubated with the extracts/fractions or pure compounds and an oxidative stress inducer (4.7 mM H_2_O_2_/3.8 mM Fe^2+^/2.5 mM EDTA) for 30 min at 37 °C. After the incubation, the samples were diluted 10 times with 0.9% NaCl. Then, 40% TCA was added on ice. The samples were incubated for 5 min and centrifuged (5 min, 2,500 rpm, 4 °C). Supernatant was discarded and 10 mM DNPH in 2 M HCl was added to the pellets. The samples were incubated for 1 h at room temperature, in the dark. 40% TCA was added on ice, the samples were incubated for 5 min and centrifuged again (5 min, 2,500 rpm, 4 °C). The supernatant was discarded. 1.5 mL of 1:1 ethanol/ethyl acetate was added to the pellets on ice and the samples were vortexed for 5 min and centrifuged (5 min, 2,500 rpm, 4 °C); this step was repeated twice. Finally, the supernatant was discarded, and the pellet was dissolved in 1 mL 6 M guanidine hydrochloride in 2 M HCl. The absorbance was measured in triplicate with a SPECTROstar Nano Microplate Reader (BMG LABTECH, Germany) at 375 nm. The levels of carbonyl groups were calculated with a molar extinction coefficient (ε = 22,000 M^−1^ cm^−1^) and expressed as nmol carbonyl groups/mg of plasma protein or nmol carbonyl groups/10^8^ of blood platelets.

### 2.11 Protein thiol measurement (in blood platelets and plasma)

The levels of thiol groups were measured with a method based on Ellman’s reagent (5,5′-dithio-bis-(2-nitrobenzoic acid), DTNB) as described earlier (Sławińska et al., 2023; 2024). Plasma or blood platelets were incubated with extracts/fractions or pure compounds and an oxidative stress inducer (4.7 mM H_2_O_2_/3.8 mM Fe^2+^/2.5 mM EDTA) for 30 min at 37 °C (final concentrations: 0,5, 5, and 50 μg/mL). A negative control (without H_2_O_2_/Fe^2+^, and extract/fraction/pure compound) with 0.9% NaCl was set up. After the incubation 20 μL of the samples were added to a 96-well plate in triplicate. 20 μL of 10% SDS in 10 mM phosphate buffer (pH 8) and 160 μL of 10 mM phosphate buffer (pH 8) were added to all samples. The absorbance was measured with a SPECTROstar Nano Microplate Reader (BMG LABTECH, Germany) at 412 nm (A_0_). Afterward, 16.6 μL of 10 mM DTNB in 10 mM phosphate buffer (pH 8) was added to the samples (16.6 μL of 10 mM phosphate buffer (pH 8) was added to the blank sample instead of DTNB). The plate was covered with parafilm and incubated at 37 °C for 1 h. Afterward, the absorbance was read again (A_1_). The levels of thiol groups were calculated with a molar extinction coefficient (ε = 13,600 M^−1^ cm^−1^) and expressed as nmol thiol groups/mg of plasma protein or nmol thiol groups/10^8^ of blood platelets.

### 2.12 Total Thrombus-formation Analysis System (T-TAS; PL-chip) (in whole blood)

The PL-chip of the T-TAS can measure the thrombus formation process in semi-physiological conditions. Full blood is pushed through a chip coated with type I collagen. Platelets are activated by collagen and 1,500/s shear stress, obstructing the flow path. Thrombus formation is monitored by measuring the pressure inside the PL-chip. The BAPA anticoagulant which was used to collect the blood for this assay blocks secondary hemostasis to allow for the assessment of only primary hemostasis. First, the tested extracts or fractions were added to full blood at final concentrations of 0, 5, 5, and 50 μg/mL. A control sample was set up, with 0.9% NaCl added instead of the extracts or fractions. The samples were incubated at 37 °C for 30 min. Then, 320 μL of the sample was added to the PL chip, and the pressure was recorded using the T-TAS for 10 min or until the time of occlusion (when the pressure reached 60 kPa). The results were recorded as the AUC_10_ (area under the curve), which is the area under the flow pressure curve recorded for 10 min after the test starts. The AUC_10_ depicts the growth, stability, and intensity of thrombus formation. The data were represented as the % of the control (a sample without extract or fractions). A more detailed description of this assay can be found in other papers (Hosokawa et al., 2011; Kaikita et al., 2019).

### 2.13 Statistical analysis

Statistical analysis was carried out with Statistica 10 (StatSoft 13.3, TIBCO Software Inc. Palo Alto, CA, United States). The distribution of data was checked with Shapiro-Wilk test. The homogeneity of variance was checked with Levene’s test. If the data had normal distribution and homogenous variance, one-way ANOVA with Tukey’s *post hoc* test was used to assess the differences between groups; otherwise, Kruskal-Wallis test was used. The results are presented as means ± SD or medians and interquartile ranges. The results were considered significant at p < 0.05. Dixon’s Q-test was used to eliminate uncertain data.

## 3 Results

### 3.1 LC-ESI/LTQOrbitrap/MS/MS profiling of L. arborea stalks and leaves extracts and leaves-derived fractions

LC-ESI/LTQ-Orbitrap/MS/MS analysis of the stalks (LaS) and leaves (LaL) extracts of *L. arborea*, together with the leaves-derived fractions (LaLA–LaLC), allowed the tentative identification of fourteen metabolites based on accurate mass measurements, elemental composition, retention times, and diagnostic MS/MS fragmentation patterns (Table 1). The identified compounds belonged to different phytochemical classes, including flavonoids (**1**, **3**, **4**, **6**, **9**, **12**, and **13**), a phenolic derivative (**2**), a fatty acid (**7**), four oxygenated fatty acids (**5**, **10**, **11**, and **14**), and a diterpene glycoside (**8**).

**TABLE 1:** Retention times (t_*R*_), Δ ppm, molecular formula, [M-H]^-^, MS/MS values of compounds occurring in *L. arborea* leaves (LaL), fractions (LaLA, LaLB, LaLC), and stalks (LaS) identified by high resolution LC-ESI/LTQOrbitrap/MS/MS (negative ion mode).

n°	t_*R*_ (min)*	Compound	Δ ppm	[M-H]^-^	Formula	MS/MS	LAL	LalA	LalB	LalC	LAS
**1**	10.10	apigenin-6,8-di-C-glycoside	0.25	593.1502	C_27_H_30_O_15_	473.1087, 383.0769, 353.0665	X	X	X	X	X
**2**	10.16	coumaroyl-*O*-glucaric acid	1.06	355.0663	C_15_H_16_O_10_	209.0300, 191.0199	X	X	X	X	X
**3**	10.58	rutin	1.02	609.1456	C_27_H_30_O_16_	301.0495	X	X	X	X	X
**4**	10.93	luteolin-7-*O*-β-glucopyranoside	1.10	447.0927	C_21_H_20_O_11_	285.0403	X	X	X	X	X
**5**	11.49	oxylipin	1.35	343.2112	C_18_H_32_O_6_	229.1445, 171.1029	---	---	---	X	X
**6**	11.70	apigenin-7-*O*-β-glucopyranoside	1.38	431.0979	C_21_H_20_O_10_	269.0451	X	X	X	X	X
**7**	12.36	azelaic acid	6.06	187.0976	C_9_H_16_O_4_	169.0863, 125.0978	X	X	X	X	X
**8**	12.86	steviolboside	0.86	641.3173	C_32_H_50_O_13_	479.2645, 317.2118	X	X	X	X	X
**9**	14.76	luteolin	3.21	285.0403	C_15_H_10_O_6_	241.0505, 175.0400, 151.0040	X	X	X	X	X
**10**	15.56	oxylipin	3.21	327.2176	C_18_H_32_O_5_	291.01963, 229.1443, 171.1028	X	X	X	X	X
**11**	16.48	oxylipin	2.73	329.2331	C_18_H_34_O_5_	293.2119, 229.1443, 171.1028	X	X	X	X	X
**12**	16.85	apigenin	2.79	269.0452	C_15_H_10_O_5_	225.0559, 201.0556	X	X	X	X	X
**13**	17.25	chrysoeriol	2.69	299.0558	C_16_H_12_O_6_	284.0325, 151.0052	X	X	X	X	X
**14**	17.60	dihydroxypalmitic acid	2.42	287.2224	C_16_H_32_O_4_	269.2121, 241.2187	X	---	X	**X**	X

Legend: * t_*R*_ derived from LC-HRMS analysis of extract LAL (leaves).

X = detectable (Normalized Level >1.0 × 10^4^); --- = not detectable.

Flavonoids represented the predominant class of secondary metabolites, occurring mainly as O-glycosides. Compound **1** (*m/z* 593.1502, t_*R*_ 10.10 min) was tentatively identified as apigenin-6,8-di-C-glycoside. In particular, its MS/MS spectra was characterized by the presence of the diagnostic product ions typical of di-C-glycosyl flavones at *m/z* 473.1087 ([M-H-120]^-^), 383.0769 ([M-H-210] ^-^), and 353.0665 ([M-H-240] ^-^), along with the absence of the aglycone product ion, supporting its assignment as a di-C-glycoside [https://doi.org/10.1002/rcm.10097]. Compound **3** (*m/z* 609.1456, t_*R*_ 10.58 min) was tentatively assigned as rutin based on the characteristic neutral loss of the rutinoside unit (308 Da), along with product ion corresponding to quercetin aglycone ion at *m/z* 301.0495 (Rahmani Samani et al., 2024) (Table 1).

Among the *O*-glycosylated flavonoids, compound **4** (*m/z* 447.0927, t_*R*_ 10.93 min) exhibited a neutral loss of 162 Da corresponding to a hexose residue, producing the aglycone ion at *m/z* 285.0403 ([luteolin−H]^-^), and was tentatively identified as luteolin-7-*O*-β-glucopyranoside (Rahmani Samani et al., 2024). Similarly, compound **6** (*m/z* 431.0979, t_*R*_ 11.70 min) generated the diagnostic fragment ion at m/z 269.0451 following the loss of a hexose unit, allowing its assignment as apigenin-7-*O*-β-glucopyranoside (Rahmani Samani et al., 2024) (Table 1).

Three flavonoid aglycones were also detected. Compound **9** (*m/z* 291.0196, t_*R*_ 14.76 min) was identified as luteolin, showing characteristic fragment ions at *m/z* 241.0505, 175.0400, and 151.0040, generated through Retro-Diels–Alder (RDA) cleavage of the flavone C-ring (Benavides et al., 2006; Rahmani Samani et al., 2024).

Compound **12** (*m/z* 269.0452, t_*R*_ 16.85 min) was assigned as apigenin based on the fragment ions at *m/z* 225.0559 and 201.0556 (Rahmani Samani et al., 2024), whereas compound **13** (*m/z* 299.0558, t_*R*_ 17.25 min) was identified as chrysoeriol through its characteristic loss of a methyl radical (*m/z* 284.0325, [M − H − 15]^-^) together with the diagnostic fragment at *m/z* 141.1872 (Noreen et al., 2021) (Table 1).

In addition to flavonoids, compound **2** (*m/z* 355.0663, t_*R*_ 10.16 min) was tentatively identified as coumaroyl-*O*-glucaric acid, based on the fragment ions at m/z 209.0300 and 191.0199 corresponding to the sequential loss of coumaric acid and water (Lorenz et al., 2008). Compound **7** (*m/z* 187.0976, t_*R*_ 12.36 min) was assigned as azelaic acid, showing a dominant product ion at *m/z* 125.0978 resulting from decarboxylation and cleavage of the dicarboxylic acid chain (Malfa et al., 2026) (Table 1).

A diterpene glycoside was also detected. MS/MS spectrum of compound **8** (*m/z* 641.3173, t_*R*_ 12.86 min) showed product ions ascribable to the sequential losses of sugars, leading to the formation of an intermediate ion at *m/z* 479.2645 ([M-H-162] ^-^) and the aglycone-related ion at *m/z* 317.2118, supporting its tentative identification as steviolbioside (Noreen et al., 2021) (Table 1).

Finally, various oxygenated fatty acid derivatives were identified. The analysis of LC-ESI/LTQOrbitrap/MS/MS spectra acquired for **5**, **10**, **11** highlighted the typical fragmentation patterns of oxylipins, a class of hydroxyl fatty acids differing by the degree of unsaturation and number of hydroxyl groups (Table 1) and derived from the oxidative metabolism of essential PUFA, such as α-linolenic acid (ALA, 18:3ω-3) and linoleic acid (LA, 18:2ω-6) (Lorenz et al., 2008). In particular, MS/MS spectra of compounds **5**, **10**, **11** were all characterized by the presence of the diagnostic product ion at *m/z* 171, corresponding to the shortened acyl chain having the carboxyl group as COO^−^ and an aldehyde as end-group originated from the rearrangement of the hydroxyl function at C9 and subsequent cleavage of the C9–C10 bond. Compound **14** (*m/z* 287.2224) generated fragment ions at *m/z* 269.2121 and 241.2187, corresponding to dehydration and cleavage of the C16 fatty acid backbone, respectively. This pattern suggests the putative identification as dihydroxypalmitic acid (Lorenz et al., 2008).

### 3.2 Identification and structural elucidation of the purified compounds (**1**, **4**, **6**, **9**, and **12**)

To unequivocally assign the structures of the major constituents detected by LC-ESI/LTQ-Orbitrap/MS/MS analysis, the crude extract of *L. arborea* leaves and the most representative chromatographic fractions were subjected to repeated chromatographic separations using a combination of silica gel and reversed-phase chromatographic techniques. This phytochemical investigation led to the isolation of five major metabolites, corresponding to compounds **1**, **4**, **6**, **9**, and **12** (Figures 1–3). Their structures were elucidated through detailed analysis of one- and two-dimensional NMR spectra and by comparison of the obtained spectroscopic data with those reported in the literature. Accordingly, compounds **1**, **4**, **6**, **9**, and **12** were identified as apigenin-6,8-di-C-glucopyranoside (Siciliano et al., 2004), luteolin-7-*O*-β-glucopyranoside (Siciliano et al., 2004), apigenin-7-*O*-β-glucopyranoside (Rahmani Samani et al., 2024), luteolin (Siciliano et al., 2004), and apigenin (Siciliano et al., 2004), respectively (Figure 3). The isolation of these metabolites enabled the unequivocal assignment of the corresponding chromatographic peaks.

**FIGURE 1:**
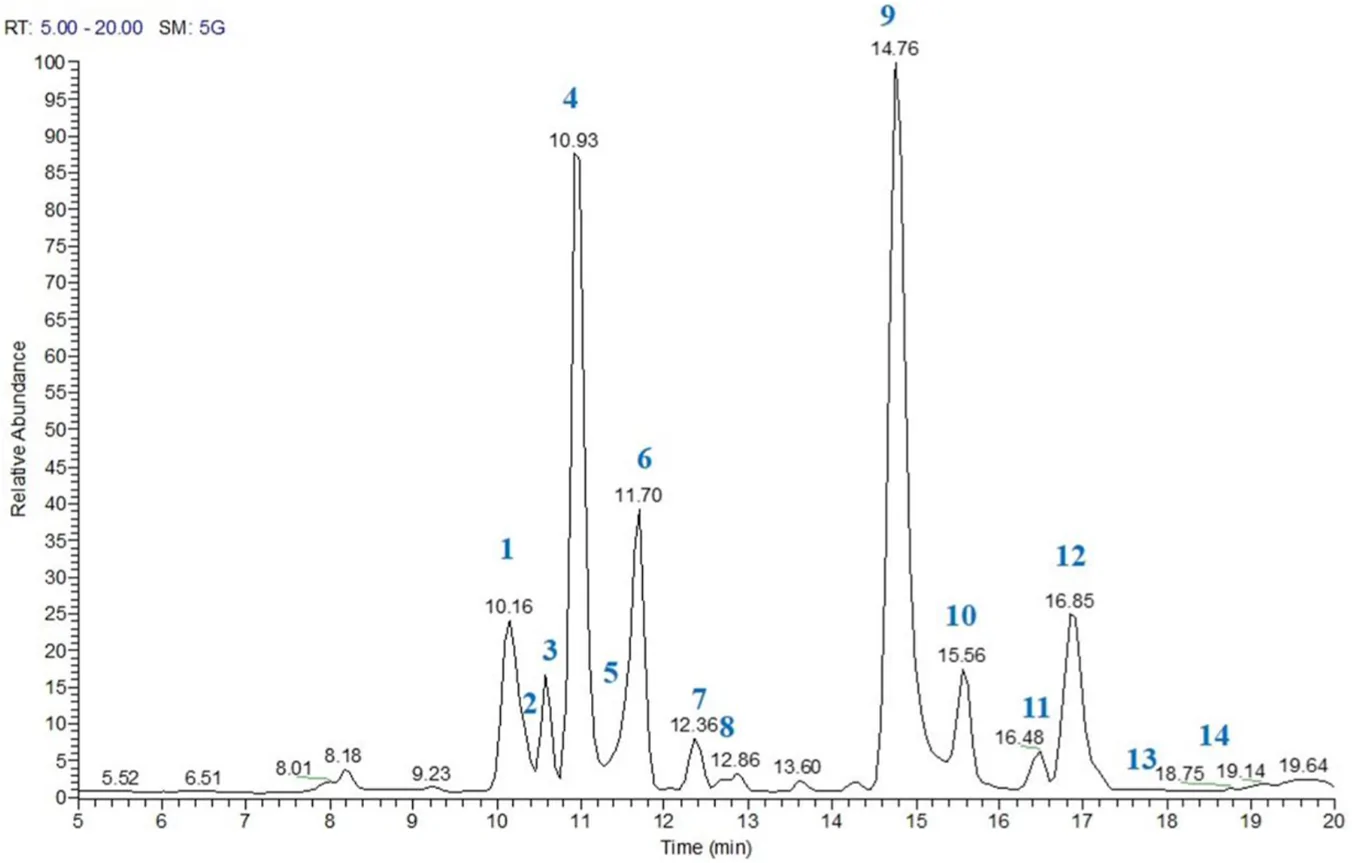
LC-ESI/LTQOrbitrap/MS/MS profile (negative-ion mode) of the MeOH extract of *L*. *arborea* leaves (LaL).

**FIGURE 2:**
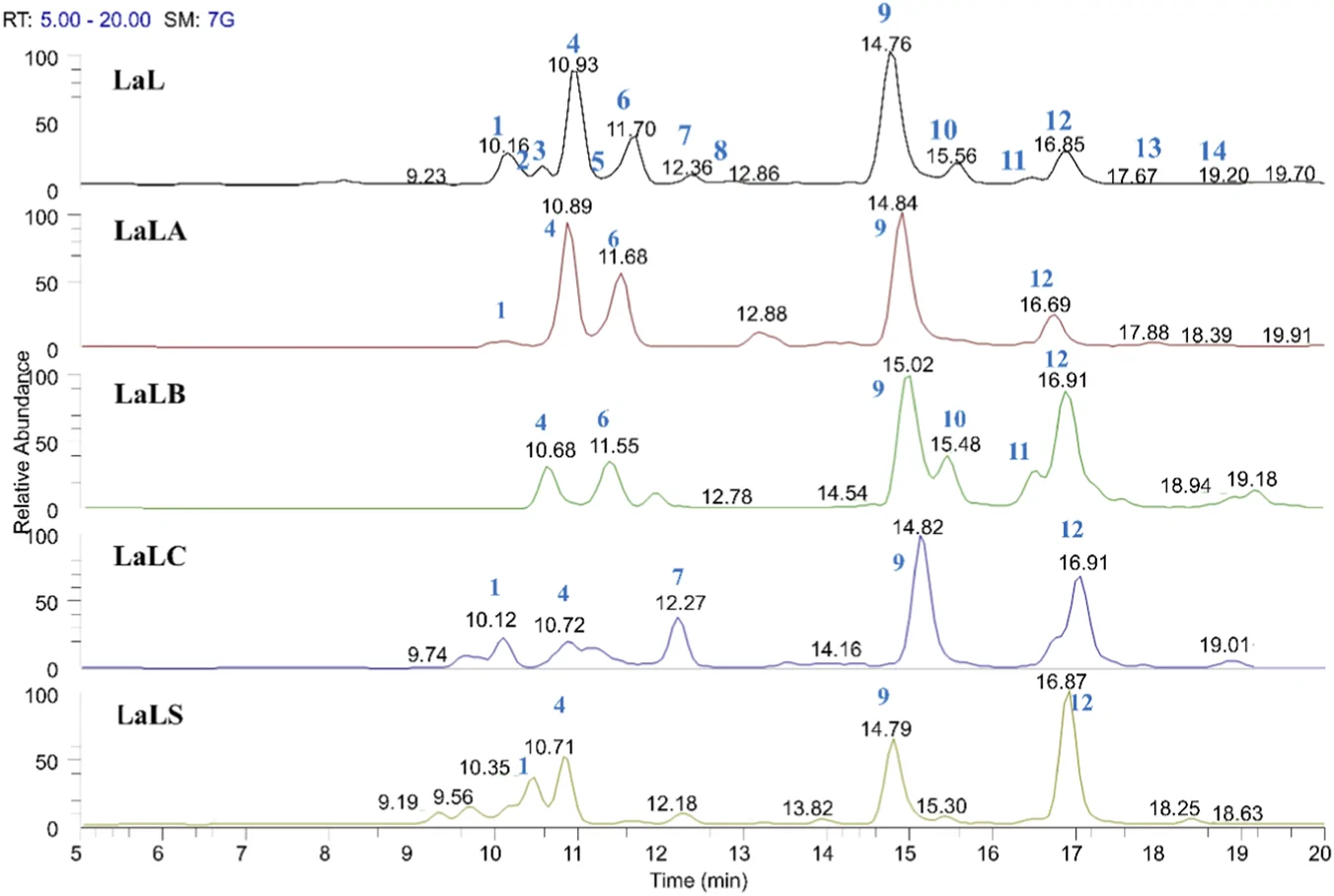
LC-ESI/HRMS/MS profiles acquired in negative-ion mode for LaL, LaLA, LaLB, LaLC, and LaS.

**FIGURE 3:**
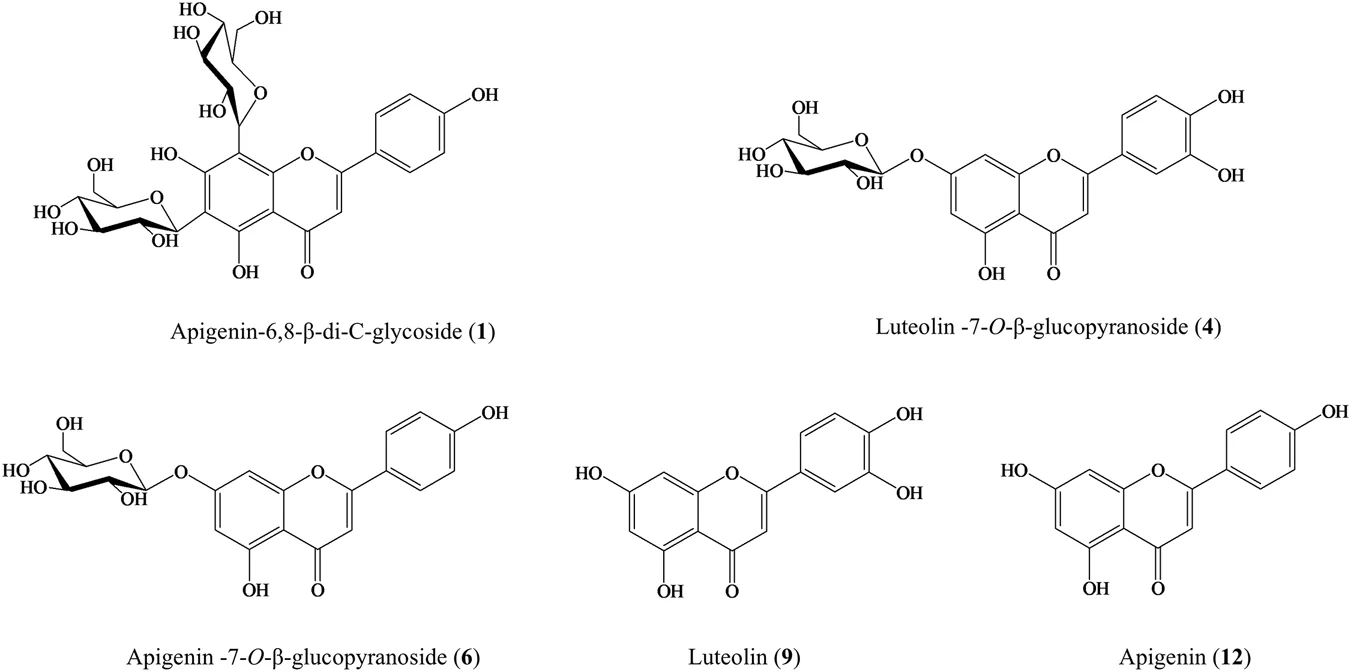
Specialized metabolites isolated from the leaf extract of *L. arborea*.

Comparison of the metabolite profiles showed that most compounds were detected in all five samples. In detail, compounds **1**–**4**, and **6**–**13** were consistently identified in all extracts. In contrast, differences were observed for compounds **5**, and **14**. Compound **5**, tentatively annotated as an oxylipin, was detected only in LalC and LaS. Compound **14**, putatively identified as dihydroxypalmitic acid, was detected in all samples except LalA.

### 3.3 Biomarkers of oxidative stress in blood platelets and plasma

The extracts and fractions from *L. arborea* did not appear to change the level of tested biomarkers of oxidative stress in plasma and blood platelets in a model without an oxidative stress inducer (data not presented). On the other hand, three used fractions (40%, 60%, and 80%) from the leaves inhibited plasma and blood platelet peroxidation induced by H_2_O_2_/Fe^2+^, but this action was not always statistically significant (Figures 4A,B). For example, the highest used concentration (50 μg/mL) of 40%, 60%, and 80% fraction significantly inhibited blood platelet lipid peroxidation by about 60% (Figure 4B).

**FIGURE 4:**
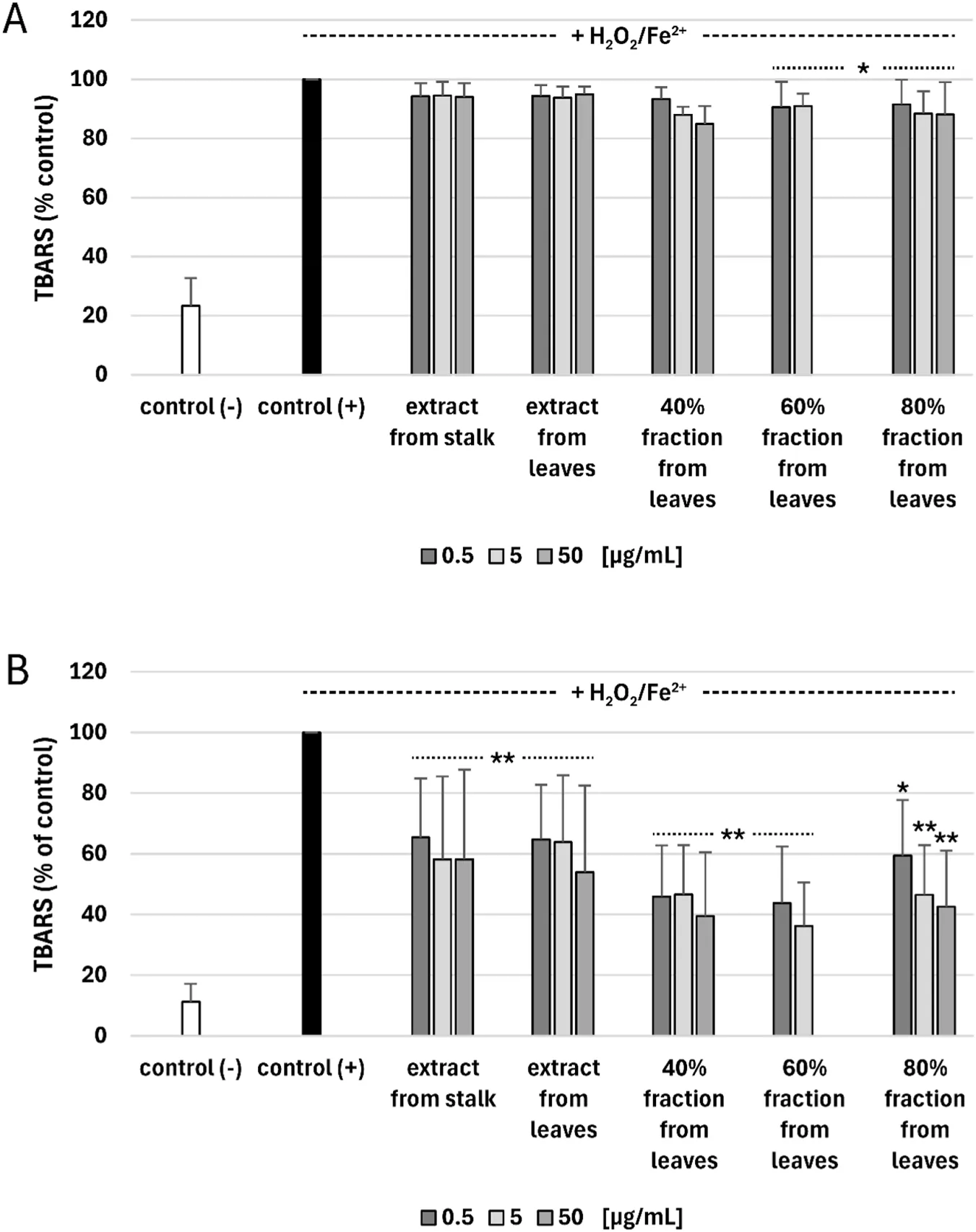
The effect of two extracts and three fractions from *L. arborea* (0.5–50 μg/mL) on the level of thiobarbituric acid reactive substances (TBARS) in human plasma treated with an oxidative stress inducer (Fe^2+^/H_2_O_2_) (n = 6) **(A)**, and in human blood platelets treated with an oxidative stress inducer (Fe^2+^/H_2_O_2_) (n = 6–10) **(B)**. Control (+) and test samples were incubated (30 min, 37 °C) with Fe^2+^/H_2_O_2_. The results are presented as % of control (+). The data are expressed as means ± SD. One way-ANOVA with Tukey’s test was used to assess the differences between the control and the test samples. The results were considered significant at p < 0.05 (*p < 0.05, **p < 0.01). The difference between control (−) and control (+) was statistically significant (p < 0.001).

Plasma protein carbonylation induced by H_2_O_2_/Fe^2+^ was reduced in the presence of all used extracts and fractions, but this process was not always statistically significant (Figure 5A). Only two concentrations of the stalks extract (5 and 50 μg/mL), two concentrations of the 80% leaves fraction (5 and 50 μg/mL), two concentrations of the 60% fraction (0.5 and 5 μg/mL), and the highest concentration (50 μg/mL) of the 40% fraction from the leaves significantly inhibited plasma protein carbonylation by about 30% (Figure 5A). In addition, both the extracts and three tested fractions (5 μg/mL) inhibited blood platelet protein carbonylation induced by H_2_O_2_/Fe^2+^. For example, 60% and 80% fractions from the leaves significantly reduced this process by about 30% and 20%, respectively (Figure 5B).

**FIGURE 5:**
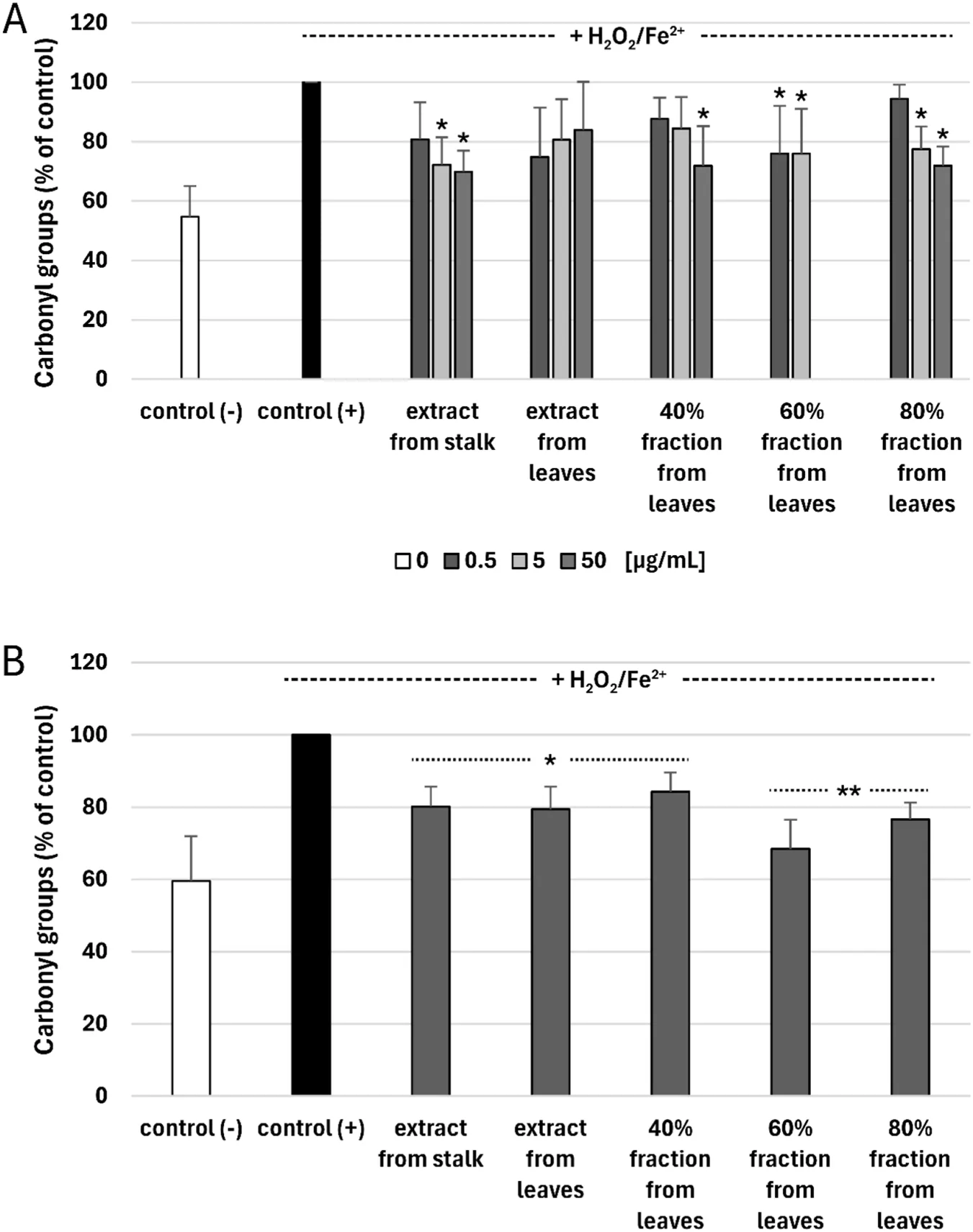
The effect of two extracts and three fractions from *L. arborea* (for plasma: 0.5–50 μg/mL, and for blood platelets: 5 μg/mL) on the level of carbonyl (CO) groups in human plasma treated with an oxidative stress inducer (Fe^2+^/H_2_O_2_) (n = 6) **(A)**, and on the level of carbonyl (CO) groups in blood platelets treated with an oxidative stress inducer (Fe^2+^/H_2_O_2_) (n = 6) **(B)**. Control (+) and test samples were incubated (30 min, 37 °C) with Fe^2+^/H_2_O_2_. The results are presented as % of control (+). One way-ANOVA with Tukey’s test was used to assess the differences between the control and the test samples. The results were considered significant at p < 0.05 (*p < 0.05, **p < 0.01). The difference between control (−) and control (+) was statistically significant (p < 0.01).

None of the tested concentrations of extract from the stalks had any effect on the oxidation of thiol groups in plasma treated with H_2_O_2_/Fe^2+^ (Figure 6A). However, two concentrations (5 and 50 μg/mL) of the leaves extract and three leaves fractions inhibited thiol oxidation induced by H_2_O_2_/Fe^2+^ in plasma (Figure 6A). Moreover, only the leaves extract (5 μg/mL) and 80% fraction from the leaves (5 μg/mL) significantly inhibited thiol group oxidation stimulated by H_2_O_2_/Fe^2+^ in blood platelets (Figure 6B).

**FIGURE 6:**
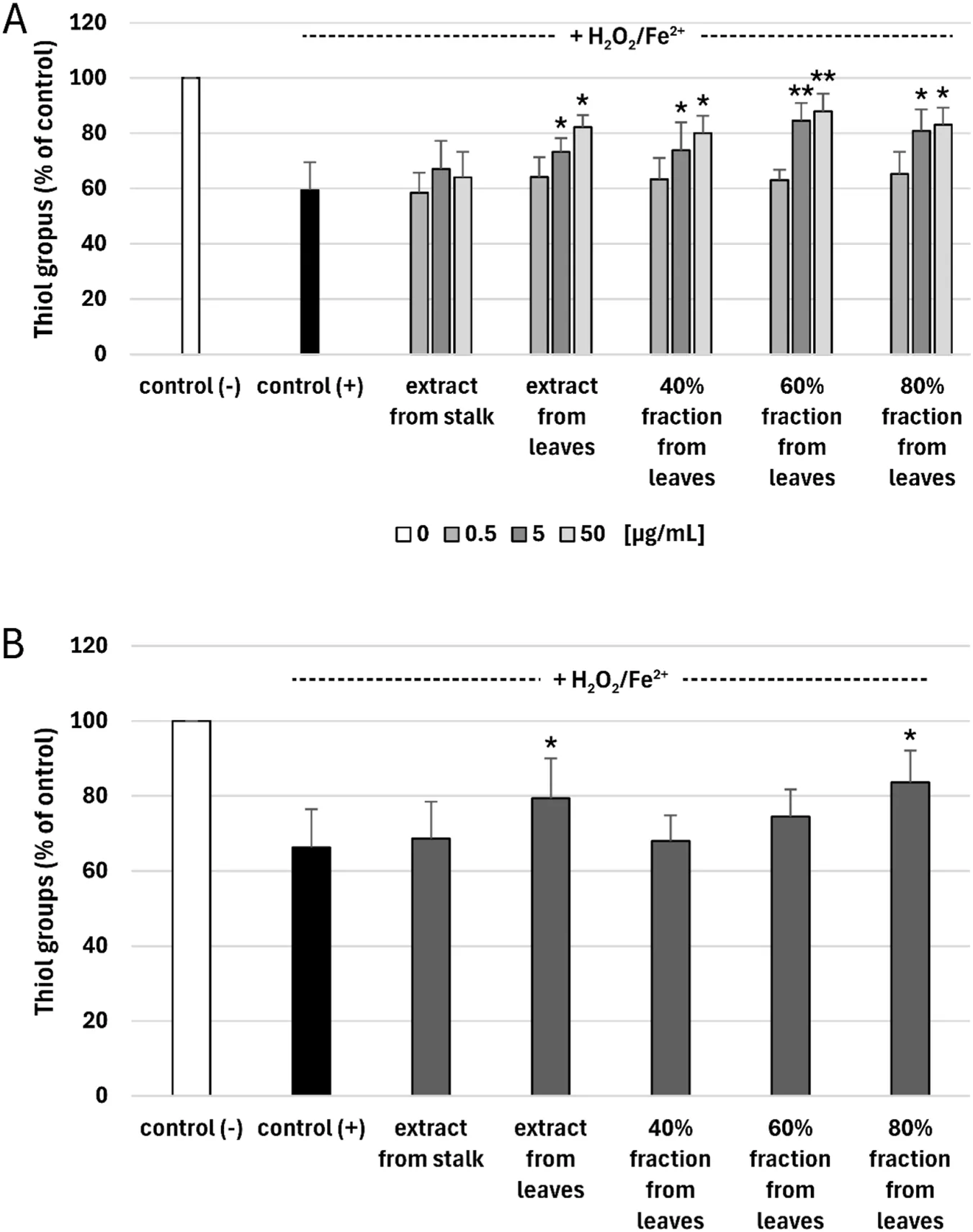
The effect of two extracts and three fractions from *L. arborea* (for plasma: 0.5–50 μg/mL, and for blood platelets: 5 μg/mL) on the level of thiol groups in human plasma treated with an oxidative stress inducer (Fe^2+^/H_2_O_2_) (n = 6) **(A)**, and on the level of thiol groups in blood platelets treated with an oxidative stress inducer (Fe^2+^/H_2_O_2_) (n = 6) **(B)**. Control (+) and test samples were incubated (30 min, 37 °C) with Fe^2+^/H_2_O_2_. The results are presented as % of control (+). One way-ANOVA with Tukey’s test was used to assess the differences between the control and the test samples. The results were considered significant at p < 0.05 (*p < 0.05, **p < 0.01). The difference between control (−) and control (+) was statistically significant (p < 0.01).

### 3.4 Total Thrombus-formation Analysis System (T-TAS; PL-chip) (using whole blood)

Only the 40% fraction from the leaves at the two highest concentrations (5 and 50 μg/mL) significantly increased the AUC_10_ value measured by T-TAS in whole blood. The AUC_10_ parameter was increased by approximately 20% (Figure 7). Aspirin (as reference anti-platelet drug, 50 μg/mL) demonstrated anti-platelet activity by significantly prolonging the time of occlusion (p < 0.05) – the AUC_10_ value was decreased by approximately 50% (data not shown).

**FIGURE 7:**
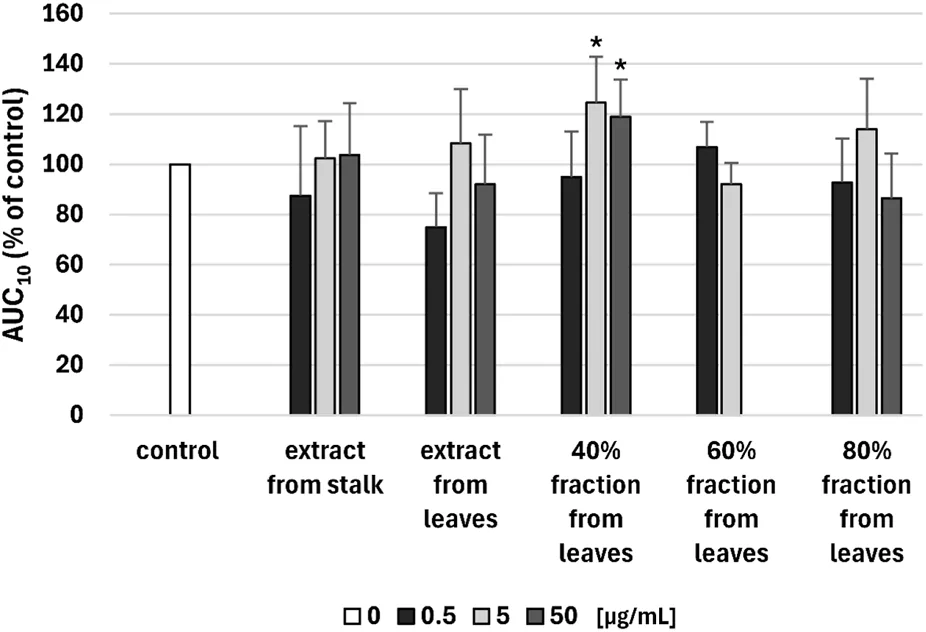
The effect of two extracts and three fractions from *L. arborea* (0.5–50 μg/mL) on platelet plug formation in whole blood (n = 4–5). The samples were analyzed with T-TAS PL-chip, at the shear stress rates of 1,500/s. The results are calculated as AUC_10_ (area under the curve). In the graphs, AUC_10_ is expressed as a percentage of the control sample (blood without the tested extract/fraction). The data are expressed as means ± SD. The results were considered significant at p < 0.05 (*p < 0.05).

### 3.5 A comparison of the biological activity of various extracts, fractions, and chemical compounds

A comparison of the effects of two extracts (from leaves and stalks) and three fractions (40%, 60%, and 80%) from the leaves at the concentration of 5 μg/mL on selected biomarkers of oxidative stress in blood platelets and plasma treated with H_2_O_2_/Fe^2+^ is demonstrated in Table 2. We observed that the 80% fraction from the leaves had stronger antioxidant potential than two extracts (from leaves and stalks) and two other fractions (40% and 60%) from the leaves. 80% fraction from the leaves showed significant antioxidant activity in all used models: (1) lipid peroxidation in blood platelets and plasma, (2) protein carbonylation in blood platelets and plasma, and (3) thiol groups oxidation in blood platelets and plasma. In addition, apigenin, luteolin, and their derivatives were prioritized for biological assessment owing to their high relative abundance in the *L. arborea* extract, where they were established as main specialized metabolites (see Supplementary Material, Supplementary Table S1) (Table 2).

**TABLE 2:** A comparison of the effect of two extracts and three fractions from *L. arborea* (5 μg/mL)*,* and selected chemical compounds (apigenin and its derivatives (apigenin 6-8-*C*-di-glucopyranoside, and apigenin 7-*O*-glucopyranoside), and luteolin and its derivative–luteolin 7-*O*-β-glucopyranoside) (5 μg/mL) present in the extracts and fractions on the level of biomarkers of oxidative stress in human plasma and blood platelets treated with Fe^2+^/H_2_O_2_.

Sample	Inhibition of lipid peroxidation (%)		Inhibition of protein carbonylation (%)		Increase of free thiols (%)	
	Plasma	Blood platelets	Plasma	Blood platelets	Plasma	Blood platelets
Extract from stalks (5 μg/mL)	No effect	48.9 ± 8.9 (**)	42.2 ± 12.4 (**)	15.7 ± 5.5 (*)	No effect	No effect
Extract from leaves (5 μg/mL)	No effect	46.7 ± 12.2 (**)	No effect	15.7 ± 6.3 (*)	23.2 ± 10.4 (*)	24.8 ± 11.1 (*)
40% fraction from leaves (5 μg/mL)	13.1 ± 7.7 (*)	54.7 ± 10.1 (**)	No effect	17.6 ± 5.3 (*)	24.4 ± 8.9 (*)	No effect
60% fraction from leaves (5 μg/mL)	11.9 ± 3.3 (*)	65.4 ± 15.1 (**)	21.9 ± 7.7 (*)	21.6 ± 8.0 (*)	42.2 ± 13.6 (**)	No effect
80% fraction from leaves (5 μg/mL)	14.2 ± 5.1 (*)	58.4 ± 13.2 (**)	23.4 ± 9.1 (*)	23.1 ± 5.2 (*)	36.2 ± 7.9 (*)	57.4 ± 13.2 (*)
Apigenin (5 μg/mL)	No effect	No effect	46.9 ± 12.8 (**)	33.8 ± 9.1 (*)	42.2 ± 13.2 (**)	44.5 ± 11.1 (**)
apigenin-6,8-di-C-glycoside (5 μg/mL)	34.7 ± 10.1 (*)	26.8 ± 5.9 (*)	44.9 ± 11.8 (**)	31.9 ± 10.1 (*)	28.9 ± 9.9 (*)	39.2 ± 12.9 (*)
Apigenin 7-*O*-glucopyranoside (5 μg/mL)	32.7 ± 9.9 (*)	22.8 ± 7.0 (*)	33.9 ± 8.9 (*)	29.4 ± 5.5 (*)	27.6 ± 9.5 (*)	36.8 ± 9.2 (*)
Luteolin (5 μg/mL)	17.9 ± 8.7 (*)	18.9 ± 5.8 (*)	22.8 ± 8.7 (*)	29.1 ± 7.2 (*)	19.9 ± 7.5 (*)	29.2 ± 9.9 (*)
Luteolin 7-*O*-β-glucopyranoside (5 μg/mL)	34.5 ± 9.9 (*)	22.9 ± 6.6 (*)	20.5 ± 6.9 (*)	33.1 ± 3.9 (*)	17.3 ± 6.1 (*)	29.1 ± 6.5 (*)
Ascorbic acid	14.9 ± 3.2 (*)	28.7 ± 8.1 (*)	60.5 ± 11.1 (**)	29.9 ± 5.9 (*)	No effect	No effect

Ascorbic acid was used as a reference antioxidant (*p < 0.05; **p < 0.01).

## 4 Discussion

### 4.1 Bioactive metabolites of *L. arborea*: phytochemical features and nutraceutical relevance

The metabolite profile of *L. arborea* was dominated by flavonoids, mainly *O*-glycosylated flavones, confirming that this class represents the major specialized metabolites of the genus *Leptadenia*. The occurrence of both glycosylated flavonoids and their aglycones, particularly apigenin and luteolin derivatives, reflects different biosynthetic and storage forms, as glycosylation enhances metabolite stability, solubility, and intracellular accumulation.

Most of the flavonoids identified in this study have previously been described in other *Leptadenia* species, supporting the conservation of flavonoid biosynthesis within the genus. These flavonoids are well known for their antioxidant and anti-inflammatory properties (Cerulli et al., 2021) and contribute to the biological activity of *L. arborea* extracts. Among the identified metabolites, compounds **4**, **6**, **9**, **12**, and **13** were previously reported in *L. arborea* (El-Moghazy et al., 1981). Compounds **1** (apigenin-6,8-di-C-glycoside) and **3** (rutin) were previously reported in the leaves of *L. hastata* (Zhang et al., 2024) and *L. reticulata* (Mohanty et al., 2017), respectively, and therefore are reported herein for the first time in *L. arborea*.

Given their well-established antioxidant and anti-inflammatory activities, these metabolites are likely to contribute to the biological properties of *L. arborea* extracts (Ramzani Ghara et al., 2021).

Regarding the other identified metabolites, compounds **2** (coumaroyl-*O*-glucaric acid) and **8** (steviolbioside) are reported herein for the first time in the genus *Leptadenia*.

Lipid-derived metabolites further enriched the phytochemical profile of *L. arborea*. Compound **7** (azelaic acid) was previously reported in the leaves of *L. reticulata* (Barot et al., 2026), whereas the polar lipids (compounds **5**, **10**, **11**, and **14**) are described here for the first time in the genus *Leptadenia*. Oxylipins and azelaic acid are recognized as plant signaling molecules involved in stress responses and have also attracted considerable interest because of their antioxidant, anti-inflammatory, and immunomodulatory activities (Garofalo et al., 2024).

Overall, this study expands the phytochemical knowledge of *L. arborea* by confirming the presence of metabolites previously reported in other *Leptadenia* species and by describing, for the first time in the genus, steviolbioside, coumaroyl-*O*-glucaric acid, and polar lipid derivatives. The predominance of flavonoids, together with phenolic acid derivatives and oxygenated fatty acids, provides a phytochemical basis for the biological activities of *L. arborea* extracts and supports their potential as a source of nutraceutically relevant bioactive compounds.

### 4.2 Isolation and structural characterization validate LC-ESI/LTQOrbitrap/MS/MS - based metabolite identification

The isolation and spectroscopic characterization of the major flavonoids confirmed the metabolite annotation obtained by LC-ESI/LTQOrbitrap/MS/MS, validating the tentative assignments based on accurate mass measurements and fragmentation patterns. Moreover, a detailed NMR analysis enabled the unequivocal determination of the glycosylation positions and the identity of the sugar moieties attached to the flavonoid aglycones, providing definitive structural confirmation of the isolated compounds. In particular, the availability of purified reference compounds permitted the unambiguous correlation between retention times, MS/MS fragmentation profiles, and molecular structures, thereby minimizing the risk of misidentification arising from the occurrence of isomeric flavonoid glycosides, which often display highly similar mass spectra. Furthermore, the identified compounds reveal that *L. arborea* leaves are characterized by a flavonoid profile dominated by apigenin- and luteolin-derived metabolites, occurring both as glycosylated derivatives and as their corresponding aglycones. The structural confirmation of these representative metabolites therefore provides a robust chemical framework for interpreting the complete metabolomic profile of *L. arborea* and constitutes a solid basis for correlating its phytochemical composition with the biological activities investigated in the present study.

### 4.3 The biological activity of various extracts, fractions, and chemical compounds isolated from *L. arborea*

Reactive oxygen species (ROS), which include free radicals (e.g., hydroxyl, superoxide, and peroxyl radicals) and non-radicals (e.g., hydrogen peroxide), play an important role in many cellular processes, such as pathogen elimination, gene expression, or redox signaling (Senoner and Dichtl, 2019; Demirci-Çekiç et al., 2022). However, a lack of balance between ROS production and the antioxidant defense capability can lead to oxidative stress, which can damage macromolecules and cause cell death (Fuentes et al., 2017; Demirci-Çekiç et al., 2022). ROS are produced mainly due to oxidative processes of aerobic systems and nicotinamide adenine dinucleotide phosphate hydrogen (NADPH) oxidase activity, or by environmental stressors like pollutants and ultraviolet radiation (UV) (Kashyap et al., 2022). The hydroxyl radical, which is produced by the reaction of H_2_O_2_ and transition metal ions, is especially reactive and damaging.

It has been established that oxidative stress plays a significant role in the development of many diseases, including cardiovascular disease, cancer, Parkinson’s disease, and Alzheimer’s disease (Senoner and Dichtl, 2019; Demirci-Çekiç et al., 2022). It is evident that oxidative stress can disrupt hemostasis by modulating blood platelet activation, coagulation process, and fibrinolysis (Violi and Pignatelli, 2012). One of the mechanisms by which ROS can affect hemostasis is deactivation of nitric oxide (NO), an important regulator of endothelium and platelet function (Gkaliagkousi et al., 2009). NO also inhibits platelet adhesion and aggregation and its deficiency can lead to a higher risk of thrombotic events (Fuentes et al., 2017). Moreover, ROS are produced by blood platelets upon activation and are essential for their functioning; they act as second messengers through calcium mobilization, interaction with arachidonic acid, or nitric oxide inactivation (Fuentes et al., 2017).

Measurement of ROS-induced modifications is the most common method to assess oxidative stress. They include protein carbonyls, advanced glycation end products, lipid oxidation products (e.g., TBARS, malondialdehyde (MDA), trans-4-hydroxy-2-nonenal (4-HNE), or isoprostanes), thiols, glutathione, or DNA/RNA oxidation. Oxidative stress can also be measured by downstream functional markers of ROS-induced damage (asymmetric dimethyl L-arginine (ADMA) or phosphorylated vasodilator-stimulated phosphoprotein (P-VASP)), markers of antioxidant defense (e.g., nuclear factor (erythroid-derived 2)-like 2 (NRF2), or markers of ROS generation (e.g., xanthine oxidase, myeloperoxidase) (Frijhoff et al., 2015). The oxidative stress model based on the donor of hydroxyl radicals is commonly used, but blood platelets and plasma are complex biological matrixes that contain various antioxidants. These compounds can influence the formation of hydroxyl radicals, but we have noted that exposure of human blood platelets and plasma resulted in a significantly enhanced level of oxidative stress measured by various biomarkers, including TBARS, carbonyl groups and thiol groups.

Plants are rich sources of antioxidants, which can combat oxidative stress by scavenging ROS, metal chelation, or repairing antioxidant enzymes such as superoxide dismutase (SOD), catalase (CAT), and glutathione peroxidase (GPx) (Demirci-Çekiç et al., 2022). Among different phytochemicals, phenolic compounds are especially interesting, as they often exhibit different beneficial properties, for example, antioxidant or antiplatelet activities (Młynarska et al., 2024).

Although species belonging to *Leptadenia* have special health-promoting properties (Mohanty et al., 2017), their biological activities at the molecular level remain only partially understood and scientific research on the bioactive compounds of some species, including *L. arborea,* is still limited. We reported for the first time that two extracts and three fractions from *L. arborea* differed in terms of antioxidant activity, in an experimental *in vitro* model based on crucial elements of hemostasis: plasma and blood platelets. The easiest way to explain these differences relates to the different chemical profiles of each of the used extracts and fractions. Our LC-MS analysis showed that various phenolic compounds, including apigenin and luteolin, were present in all the extracts and fractions; these compounds might be the determinants of their biological potential.

Apigenin is a flavonoid present in many fruits, vegetables, and herbs. Multiple studies have shown its anti-inflammatory, antibacterial, antiviral, antitumor, hepatoprotective, cardioprotective, and antiplatelet activities (Wu et al., 2021; Kashyap et al., 2022; Li et al., 2023). Our phytochemical analysis showed that not only apigenin, but also its derivatives can be found in *L. arborea* leaves and stalk. It is important to note, that apigenin and its derivatives are potent antioxidants (Kowalska et al., 2020). They can affect the activity of various antioxidant enzymes such as cyclooxygenase (COX-2), xanthine oxidase (XO), or nitric oxide synthase (NOS) and inhibit NO production. They interact with redox signaling pathways (e.g., Nrf-2-mediated pathways), scavenge ROS, chelate transition metals, and strengthen enzymatic antioxidants (Kashyap et al., 2022). For example, in an *in vivo* study on BALB/c mice, apigenin administered intragastrically (at the dosages of 125–500 mg/kg) inhibited oxidative stress caused by doxorubicin. It increased SOD activity and glutathione (GSH) expression, at the same time reducing MDA levels. It also significantly suppressed ROS overproduction in NRK-52E (rat kidney) cells *in vitro* (Wu et al., 2021). In a study by Kim et al. (2014), apigenin derivative present in *L. arborea*, apigenin 7-*O*-glucopyranoside, showed radical-scavenging activity against 2,2-diphenyl-1-picrylhydrazyl (DPPH) (IC_50_ = 153.7 μM) and 2,2'-azino-bis(3-ethylbenzothiazoline-6-sulfonic acid (ABTS) (IC_50_ = 63.9 μM) radicals, though it did not protect HepG2 cells from tert-butyl hydroperoxide (*t*-BHP)-induced cell death (Kim et al., 2014). Other researchers also noted that apigenin and its derivatives, including (3, 4, 5, 7-tetrahydroxy flavone), possess antioxidant activity (Kowalska et al., 2020). These results suggest that apigenin and its derivatives (for example apigenin-6, 8-di-C-glycoside or apigenin-7-*O*-β-glucopyranoside) present in *L. arborea* leaves and stalk are important contributors to the antioxidant activity of the tested extracts and fractions.

Another phenolic compound, luteolin, was also found in all the tested extracts and fractions of *L. arborea*. Luteolin, a flavonoid with two benzene rings and hydroxyl groups, shows a wide array of beneficial properties, including antioxidant, antiplatelet, anti-inflammatory, anticancer, anti-diabetes, hepatoprotective, anti-atherosclerotic, and nephroprotective activities (Kasala et al., 2016; Ye et al., 2023; Fernando et al., 2024; Zhu et al., 2024; Wan et al., 2025). In a study by Fernando et al. (2024) (Fernando et al., 2024), luteolin ameliorated oxidative stress-induced damage in lung fibroblasts by scavenging ROS, inhibiting H_2_O_2_-induced lipid peroxidation, increasing GSH levels, and enhancing the activity of SOD, CAT, GPx, and heme oxygenase-1 (HO-1). The antioxidant activity of luteolin was also studied *in vivo*. At the dose of 15 mg/kg, it inhibited lipid peroxidation in serum and lungs of rats with benzo(a)pyrene-induced lung cancer. It also restored enzymatic (SOD, CAT, GPx, glutathione reductase (GR), and glutathione-S-transferase (GST)) and non-enzymatic (GSH, vitamin E, vitamin C) antioxidants to near normal levels (Kasala et al., 2016). Luteolin 7-*O*-β-glucopyranoside also showed radical-scavenging potential. Tatli et al. (2007) report that its ortho-dihydroxy groups and glucopyranosyl unit at C-3′ position allowed it to have stronger DPPH-scavenging activity than apigenin 7-*O*-glucopyranoside and luteolin 3′-*O*-glucopyranoside (Tatli et al., 2007). Our present results in an *in vitro* model also demonstrated that luteolin and its derivatives reduce lipid peroxidation, protein carbonylation, and thiol oxidation in plasma and blood platelets treated with H_2_O_2_/Fe^2+^.

The antioxidant mechanisms of the tested extracts, fractions, and pure chemical compounds from *L. arborea* may include scavenging oxidants (H_2_O_2_/Fe^2+^ - derived hydroxyl radicals, and H_2_O_2_ itself). However, the tested extracts and fractions can contain other metabolites which might affect their antioxidant properties, or modulate other molecular pathways. Interestingly, isolated phenolic compounds are often less effective antioxidants than different plant preparations that contain a combination of compounds. This is in line with our results - tested extracts and fractions were often more effective antioxidant factors than pure phenolic compounds isolated from *L. arborea*. These results indicate that different chemical compounds present in the extracts and fractions might have synergistic properties. For the first time, our present experiments indicate that 14 identified chemical compounds, including apigenin, luteolin, and their derivatives have been identified from LalC fraction from *L. arborea* leaves which has stronger antioxidant potential than other tested fractions and extracts (containing 13–16 chemical compounds) *in vitro*. Other authors also indicate that preparations from *L. arborea* are the source of chemical compounds with antioxidant properties. Mohanty et al. evaluated lipid peroxidation inhibition capabilities of this plant by assessing the TBARS levels. Analysis of the most active fraction revealed the presence of one phenolic compound (*p*-coumaric acid) and two flavonoids (rutin and quercetin), which were determined quantitatively. The researchers observed a decrease in TBARS concentration, which revealed the potential of this plant to inhibit lipid peroxidation (Mohanty et al., 2015). However, the effects of extracts or fractions from *L. arborea* stalk and leaves on various components of hemostasis remain poorly understood. To address this gap in knowledge, we employed the T-TAS method to study blood platelet activation in whole blood, which is a more natural environment than media in which platelets are suspended after isolation. Specifically, we used the PL-chip, which allows for the evaluation of primary hemostasis by blocking secondary hemostasis. In addition, it can monitor the effectiveness of antiplatelet drugs or supplements in patients with CVDs (Mohanty et al., 2015). On the other hand, a limitation of this method is that it does not provide information about individual platelet activation pathways. For the first time, we noted that the 40% fraction from *L. arborea* leaves (5 μg/mL) has procoagulant properties *in vitro*. Other tested fractions from the leaves (60% and 80%) and two tested extracts from leaves and stalk did not show any antiplatelet or procoagulant properties. However, all used extracts and fractions from *L. arborea* are sources of compounds with anti-platelet activity. For example, apigenin has an antiplatelet effect - it competes for binding to platelet thromboxane A_2_ (TxA_2_) receptors and interferes with protease-activated receptor-1 (PAR-1), Ca^2+^, and P2Y_12_ signaling (Navarro-Núñez et al., 2008; Tamer et al., 2022). 200 μM apigenin significantly decreased platelet adhesion measured by perfusion studies. It also synergized with aspirin in the suppression of arachidonic acid pathway (Navarro-Núñez et al., 2008). In a recent study, Ye et al. (2023) (Ye et al., 2023) also reported that luteolin inhibits collagen- and convulxin-mediated platelet aggregation and adhesion, as well as integrin α_IIb_β_3_-mediated platelet activation. Molecular docking analysis showed that it can decrease the binding between collagen and glycoprotein (GP)IV. Moreover, at a dose of 35 μM/kg, luteolin significantly prolonged the time of clot obstruction of blood vessels *in vivo* (measured in a mouse model of mesenteric thrombosis induced by ferric chloride). In a study by Afifi and Abu-Dahab (2012), luteolin inhibited platelet aggregation induced by collagen (IC_50_ = 0.034 mg/mL) and adenosine diphosphate (ADP) (IC_50_ = 0.015 mg/mL) (Afifi and Abu-Dahab, 2012). Lee et al. (2023) also showed that luteolin (at the concentrations of 32 and 64 μM) inhibits platelet aggregation induced by collagen. It also significantly decreased TXA_2_ production in human platelets (Lee et al., 2023).

### 4.4 Limitations

For the first time, in our present work, we demonstrated that the tested phenolic compounds, including apigenin, luteolin, and their derivatives might be the determinates of biological potential of *L. arborea.* Despite the promising *in vitro* antioxidant properties of these compounds*,* their poor bioavailability might be an obstacle in achieving the same results *in vivo* models. On the other hand, several authors suggest that *in vitro* concentrations of phenolic compounds at or below 50 μg/mL (which were also used in the present study) correspond with physiological concentrations available after oral administration (Manach et al., 2004; 2005; Johnson, 2007). However, it is essential to analyze the concentration of circulating phenolic compounds and their metabolites, and determine if physiologically achieved concentrations correlate with those used in our *in vitro* study. Guo et al. (2017) indicate that the antioxidant properties of phenolic compounds might be enhanced by digestion (Guo et al., 2017), however each compound has to be considered individually.

### 4.5 Future perspectives

The present study showed that *L. arborea* leaves and stalk are valuable sources of bioactive compounds, but more research is needed to provide a better understanding of their antioxidant properties and determine their effectiveness *in vivo*. Future *in vitro* and *in vivo* experiments are also necessary to understand the modulation of hemostasis by the extracts or fractions from *L. arborea* and their bioactive compounds in the context of cardiovascular diseases, where oxidative stress plays an important role.

## 5 Conclusion

The present *in vitro* study provides new information about the chemical content and antioxidant activity of *L. arborea* leaves and stalk. The plant had a high flavonoid content (mostly *O*-glycosylated flavones); it also contained phenolic acid derivatives and oxygenated fatty acids. Some of the compounds detected in leaves and stalk of *L. arborea* were previously reported in other *Leptadenia* species (e.g., apigenin-6,8-di-C-glycoside and rutin), while steviolbioside, coumaroyl-*O*-glucaric acid, and polar lipid derivatives were described for the first time in the genus. The extracts and fractions from *L. arborea* did not change the level of oxidative stress biomarkers in plasma and blood platelets in a model without an oxidative stress inducer. However, different extracts and fractions from leaves and stalk of *L. arborea* decreased oxidative stress levels in plasma and blood platelets treated with H_2_O_2_/Fe^2+^ (a donor of hydroxyl radical). The 80% fraction isolated from *L. arborea* leaves was the most effective out of all the tested preparations. Moreover, 40% fraction from the leaves at the two highest concentrations (5 and 50 μg/mL) significantly increased the AUC_10_ value measured by T-TAS in whole blood. The present study showed that *L. arborea* leaves and stalk are valuable sources of bioactive compounds, but more research is needed to provide a better understanding of their antioxidant properties and determine their effectiveness *in vivo*.

## Data Availability

The datasets generated and analyzed for this study can be found in the Metabolomics Workbench repository under Study ID [8272] at the following link: https://www.metabolomicsworkbench.org/data/ds_main.php?tag=antocerulli89_20261009_032142&DATATRACK_ID=8272.
