# Supplementary material for: LC-HRMS/MS-based qualitative secondary metabolites profiling of Leptadenia arborea and evaluation of its antioxidant and hemostatic activities
Source: Front Mol Biosci. 2026 Oct 21;13:1951196. doi: 10.3389/fmolb.2026.1951196 (PMC13730956; doi:10.3389/fmolb.2026.1951196)
Supplement: Supplementary file 1 [file Table1.docx]

**Table S1.** Relative peak intensity of metabolites identified in *L. arborea* leaves (LaL), fractions (LaLA, LaLB, LaLC), and stalks (LaS) LC-ESI/LTQOrbitrap/MS/MS (negative ion mode).

|  | t*_R_* **(min)** | **Compound** | **Δ ppm** | **[M-H]^-^** | **Formula** | **LAL** | **LalA** | **LalB** | **LalC** | **LAS** |
| --- | --- | --- | --- | --- | --- | --- | --- | --- | --- | --- |
| **1** | 10.10 | apigenin-6,8-di-C-glycoside | 0.25 | 593.1502 | C_27_H_29_O_15_ | 3.89x106 | 8.16x105 | 3.28x105 | 1.06x106 | 3.32x106 |
| **2** | 10.16 | coumaroyl-*O*-glucaric acid | 1.06 | 355.0663 | C_15_H_16_O_10_ | 1.58x106 | 1.05x106 | 2.87x104 | 1.26x105 | 1.47x106 |
| **3** | 10.58 | rutin | 1.02 | 609.1456 | C_27_H_29_O_16_ | 2.61x106 | 6.15x105 | 1.24x106 | 1.30x105 | 1.30x106 |
| **4** | 10.93 | luteolin-7-*O*-β-glucopyranoside | 1.10 | 447.0927 | C_21_H_20_O_11_ | 2.59x107 | 1.94x107 | 1.99x107 | 3.99x106 | 1.49x106 |
| **5** | 11.49 | oxylipin | 1.35 | 343.2112 | C_18_H_32_O_6_ | --- | --- | --- | 1.48x106 | 2.54x106 |
| **6** | 11.70 | apigenin-7-*O*-β-glucopyranoside | 1.38 | 431.0979 | C_21_H_20_O_10_ | 8.47x106 | 5.05x106 | 6.73x106 | 1.16x106 | 3.20x105 |
| **7** | 12.36 | azelaic acid | 6.06 | 187.0976 | C_9_H_16_O_4_ | 5.60x106 | 8.60x105 | 2.26x106 | 5.51x106 | 8.32x105 |
| **8** | 12.86 | steviolboside | 0.86 | 641.3173 | C_32_H_50_O_13_ | 6.76x105 | 6.66x105 | 5.81x105 | 3.69x104 | 5.46x105 |
| **9** | 14.76 | luteolin | 3.21 | 285.0403 | C_15_H_10_O_6_ | 1.85x107 | 1.34x107 | 1.36x107 | 1.25x107 | 6.70x106 |
| **10** | 15.56 | oxylipin | 3.21 | 327.2176 | C_18_H_32_O_5_ | 3.46x106 | 5.01x105 | 3.04x106 | 1.80x105 | 7.78x105 |
| **11** | 16.48 | oxylipin | 2.73 | 329.2331 | C_18_H_34_O_5_ | 3.24x106 | 5.54x105 | 2.30x106 | 2.77x106 | 4.45x105 |
| **12** | 16.85 | apigenin | 2.79 | 269.0452 | C_15_H_10_O_5_ | 9.33x106 | 4.56x106 | 8.65x106 | 7.42x106 | 3.24x106 |
| **13** | 17.25 | chrysoeriol | 2.69 | 299.0558 | C_16_H_12_O_6_ | 6.38x105 | 5.11x105 | 5.41x105 | 4.27x105 | 5.22x105 |
| **14** | 17.60 | dihydroxypalmitic acid | 2.42 | 287.2224 | C_16_H_32_O_4_ | 7.78x105 | --- | 7.0x105 | 4.45x105 | 2.0x104 |

Legend: * t*_R_* derived from LC-HRMS analysis of extract LAL (leaves).

X = detectable (Normalized Level >1.0 × 10^4^); ---= not detectable.
